# An ethnobotanical survey on the medicinal and edible plants used by the Daur people in China

**DOI:** 10.1186/s13002-024-00695-8

**Published:** 2024-05-24

**Authors:** Yaqiong Bi, Feng Gao, Jingxia Guo, Xia Yao, Aixiang Wang, Haolin Liu, Yahong Sun, Ruyu Yao, Minhui Li

**Affiliations:** 1 Inner Mongolia Traditional Chinese & Mongolian Medical Research Institute, Hohhot, 010020 China; 2 Inner Mongolia Medical University, Hohhot, 010110 China; 3 Inner Mongolia Key Laboratory of Characteristic Geoherbs Resources Protection and Utilization, Baotou Medical College, Baotou, 014060 China; 4 Institute of Medicinal Plant Development, Chinese Academy of Medical Sciences and Peking Union Medical College, Beijing, 100193 China; 5 Inner Mongolia Institute of Traditional Chinese Medicine, Jiankang Road 11, Hohhot, 010020 Inner Mongolia China

**Keywords:** Daur, Ethnobotanical knowledge, Nutrition, Plant resource, Culture, Sustainability

## Abstract

**Background:**

The Daur people are one of the 55 minority ethnic groups in China and have lived in Northern China for 300 years. In traditional Daur medicine, medicinal and edible plants (MEPs) are utilised for health benefits and therapeutic purposes; however, related ethnobotanical knowledge is rarely reported, which is disadvantageous for the sustainable development of these MEPs.

**Methods:**

Semi-structured interviews with 122 informants, six focus group discussions, and a resource survey were conducted in a Daur minority nationality area in Inner Mongolia from 2015 to 2020, and the data statistics were analysed. In this study, we simulated a system dynamics model aimed at understanding the multiple feedback mechanisms involved in the relationships between the cultural influences and socioeconomic factors, sustainable environment, and development of MEPs.

**Results:**

A total of 52 species of MEPs were identified and relevant ethnobotanical knowledge was assessed using Daur medicinal species data from Inner Mongolia and the Xinjiang region, with the literature and Ewenki ethnic group data used for comparison. The most commonly used medicinal plant species by the Daur were found to be *Betula pendula* subsp. *mandshurica*, *Artemisia integrifolia*, *Crataegus pinnatifida*, *Saposhnikovia divaricata*, *Artemisia argyi*, and *Jacobaea cannabifolia*. The MEPs most frequently targeted the digestive and rheumatic immunity systems, as well as infectious diseases or parasitic infections and other common diseases and basic health issues. MEP knowledge was primarily limited to older generations; thus, the valuable ethnobotanical knowledge on traditional medicines must be protected from future losses.

**Conclusions:**

Our findings provide insights for future research aimed at exploiting the rich phytochemical diversity in traditional medicine and promote its use in modern lifestyles. Effective assessment and management of plant resources will lead to their application for the improvement of dietary diversity, nutrition, and health care.

**Supplementary Information:**

The online version contains supplementary material available at 10.1186/s13002-024-00695-8.

## Background

Owing to their considerable biological diversity and wide array of phytochemicals, edible plants have been an essential source of food and medicine for millennia [1, 2]. The recognition of the benefits of a plant-based diet as part of a holistic healthcare solution has gradually increased. However, the domestication of plants has led to a reduced crop diversity and a gradual shift from high to low phytochemistry [3, 4]. Health maintenance of the general population through promoting the consumption of a greater variety of nutritional plants is a significant challenge. Unlike modern medicines, traditional medicinal plants have previously been used in ways that resemble dietary interventions, with characteristics such as a complex material basis, long-term preference, and high nutritional security, in many nations and regions. Dietary interventions with medicinal plants and their derived products have been studied for many diseases, such as cancer, Alzheimer’s disease, and obesity [5–7]. The scaling of traditional medicinal plant usage will depend on the vast population knowledge, acknowledgement of safety concerns, and effectiveness of the prolonged use for diverse dietary needs [8–10].

China is one of the most biodiverse countries in the world, particularly in terms of medicinal plants [11, 12]. According to previous reports [13], 7736 species of traditional medicinal plants have been utilised by ethnic minorities in China, reflecting the diverse food, culture, distribution, and development of these different ethnic groups [14]. The Daur ethnic group is one of many minorities with ethnobotanical knowledge. Most Daur people moved to Inner Mongolia owing to war, but a small population lives in Heilongjiang and Xinjiang [15]. The Daur people have their own language but no exclusive script. Shamanism was the main religious practice of the Daur ethnic group, influencing their worldview and customs in the past, but it has almost disappeared [16]. For a long time, the Daur people have depended on wild medicinal and edible plants (MEPs) to ensure their nutritional security and reduce livelihood risks. These MEPs are a potential treasure trove of medicines and health products that are yet to be developed. However, gradual changes in the ecological environment and socioeconomic development have caused the addition and disappearance of many MEP species [17]. Therefore, it is important to catalogue knowledge of the Daur ethnic group on MEPs to conduct in-depth research that will help understand the relationships between MEPs, cultural diversity, ecological environment deterioration, and socioeconomic changes.

The urgent need for Daur medicinal plant research has been increasingly recognised by the scientific community; however, only a small proportion of available medicinal plant records describe foods used for health care. In the twentieth century, Guo et al. [18] were the first to survey the use of 14 plant medicines and 16 animal medicines by the Daur as ethnic medicines. Additionally, a study by Liu et al. [17] recorded 31 varieties of medicines, including products and materials from both plants and animals. Subsequently, Zhang et al. [19] summarised records from previous studies and laid a theoretical foundation for further investigation of medicinal plants used by the Daur ethnic group. These studies have directly documented traditional medicinal plant applications; however, no studies have investigated their mechanisms of action or inheritance. Furthermore, changes in socioeconomic, cultural, and ecological resources have affected the potential use of MEPs used by the Daur ethnic group. In this study, we aimed to: (i) investigate MEPs used by the Daur ethnic group; (ii) explore the variations in MEPs among regions inhabited by the same Daur ethnic group and the differences in MEP use between the Daur and other ethnic groups within the same region; and (iii) evaluate the effects of sustainable resources, culture, and socioeconomic factors on MEP development.

## Methods

### Study area

**Fig. 1:**
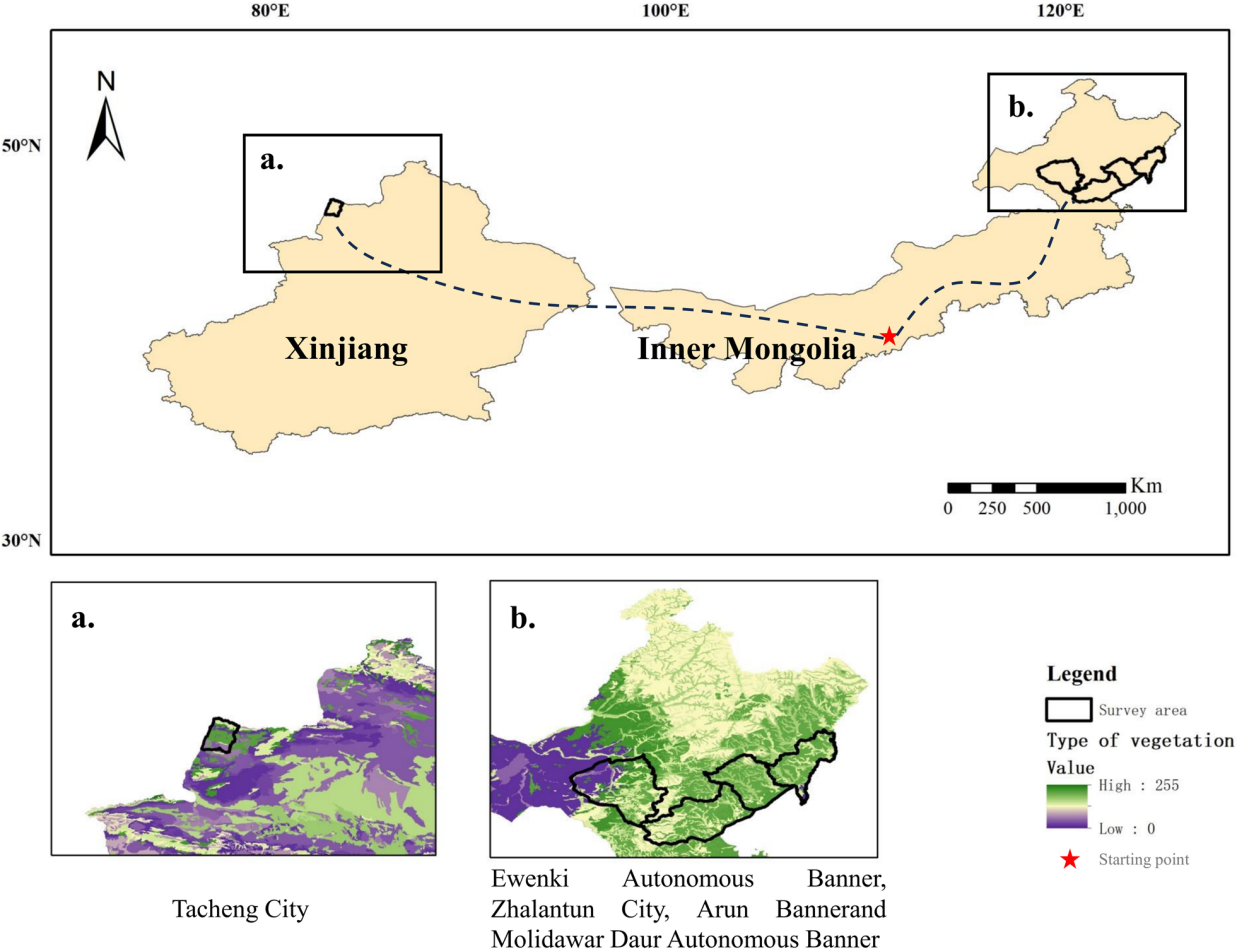
Study area map showing the Daur minority nationality area in Inner Mongolia and Xinjiang. (**a** Vegetation types in the Tacheng City of Xinjiang, **b** Vegetation types in the Ewenki Autonomous Banner, Arun Banner, Zhalantun City, and Molidawar Daur Autonomous Banner of Inner Mongolia)

The Daur group consists of a population of 132,299 according to the Seventh Population Census, of which approximately half live in Hulunbuir City, Inner Mongolia [20] within the Ewenki Autonomous Banner, Arun Banner, Zhalantun City, and Molidawar Daur Autonomous Banner [19] (Fig. 1b, Table S1). The majority of the Daur people live in the Greater Khingan Mountains within the central and eastern regions, which are predominantly located in temperate continental monsoon zones and some in cold temperate zones. Climatic characteristics of the region include long, cold winters and short, cool summers, in addition to an abundance of sunshine and little precipitation, mostly occurring between July and August. The area comprises various biome types, such as mountains, grasslands, and plateaus. In addition to the Daur ethnic group, these areas support a range of minorities, including the Mongolian [21], Ewenki [22], and other ethnic groups. The region investigated in the present study has varied topography, highly diversified flora, multiple ethnic groups, and a rich cultural diversity [20, 23].

A group of Daur people moved westward and defended their territory during the war in the late eighteenth century; these people are now distributed around Tacheng City in Xinjiang Province (Fig. 1a). The Daur and 29 other ethnic groups, including Uyghur, Kazakh, and Mongolian populations, became allies and built a new homeland. Currently, approximately 3% of the Daur population lives in Tacheng (Table S1). The ethnic gathering area is located in the Tae Basin, where resources and the environment differ greatly from those in the Daur ethnic region in Inner Mongolia. To explore the development and cultural history of Daur national medicine, we conducted a semi-structured survey among Daur healers in Inner Mongolia and the Xinjiang region and compared medicinal information. However, the information obtained in Xinjiang, especially regarding the use and dosage of MEPs, was limited and incomplete. Therefore, we did not include the Xinjiang data in our main investigation and analyses and used this information only for comparative analysis of regional differences.

### Data collection

#### Resource survey and sample collection

A team consisting of ethnobotanists, taxonomists, and Daur individuals conducted the resource surveys and sample collection. We used a combination of sample area and transect surveys to allow documentation of a wide statistical species distribution within the Daur minority nationality area in Inner Mongolia from 2015 to 2020. We did not conduct resource surveys and collections in the Xinjiang region owing to the limited information collected locally on medicinal plant uses, as most species are cultivated for food.

**Fig. 2:**
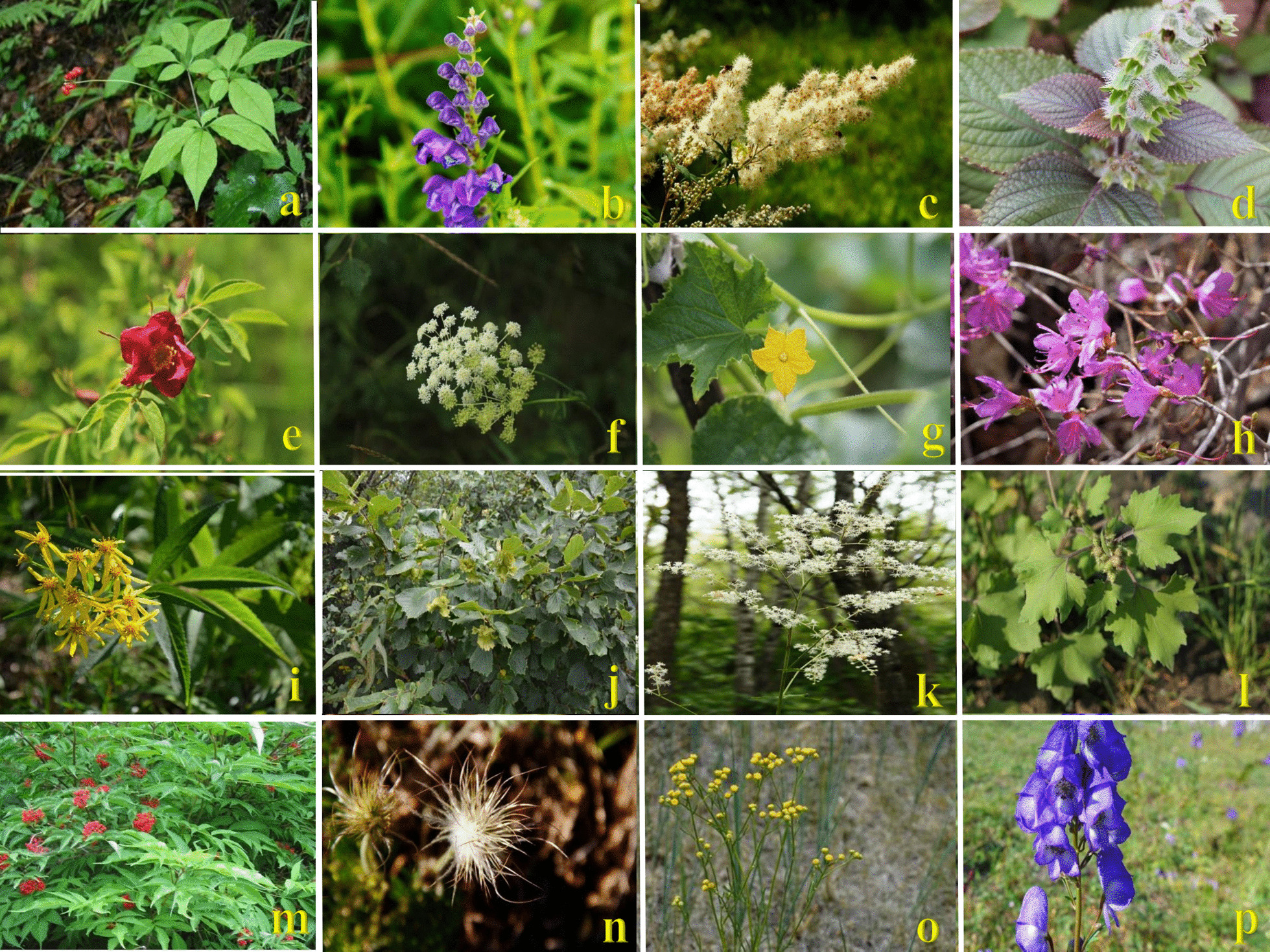
Parts of plants used in Daur medicine at study area (**a. ***Panax ginseng* C. A. Mey., **b.** *Scutellaria baicalensis* Georgi, **c.** *Sorbaria sorbifolia* (L.) A.Braun, **d.** *Perilla frutescens* (L.) Britton, **e.** *Rosa davurica* Pall., **f.** *Cicuta virosa* L., **g.** *Cucumis sativus* L., **h.*** Rhododendron dauricum* L., **i.** *Jacobaea* *cannabifolia* (Less.) E. Wiebe, **j.** *Corylus heterophylla* Fisch. ex Trautv., **k.** *Actaea dahurica* (Turcz. ex Fisch. & C.A.Mey.) Franch.., **l.** *Xanthium strumarium* L., **m.** *Sambucus williamsii* Hance, **n.** *Pulsatilla chinensis* (Bunge) Regel, **o. ***Filifolium sibiricum* (L.) Kitam., **p.** *Aconitum kusnezoffii* Rchb.)

Field investigations involved collecting various MEPs (Fig. 2), recording their habitat and location information, and taking reference photographs. Additional information on the abundance and distribution of MEPs in the study area was collected. Fieldwork was highly beneficial for the assessment and appreciation of the rich inheritance of MEP knowledge and the diversity of ethnic culture. The family and scientific names of voucher specimens were confirmed using the Plant List database (www.worldfloraonline.org). Voucher specimens of plants were prepared and deposited at the Baotou Medical College, Inner Mongolia University, China.

#### Data collection for frequently used MEPs

The data were collected from the following two types of sources: 1) interviews, focus group discussions, and field observations in the study areas and 2) publications and reviews relevant to the present study [24]. A research team conducted semi-structured interviews with 122 informants (including healers, ethnic medicine gatherers, and local villagers) using preprepared questions (Table S3). The study employed 98 questionnaires that were used to collect various types of data. Local traditional medicine practitioners, such as experts and advisors on traditional MEP knowledge and application, provided basic information on ethnomedicine and views on the development status of MEPs, whereas villagers described the effects of MEPs as part of disease treatments and the efficacy of their daily consumption.

The data collection team paid particular attention to the choice of language spoken in the interviews, noting the use of the Chinese, Mongolian, and Daur languages by the interviewees. Prior to the interviews, we obtained oral consent from the interviewees that the information provided could be made public. Those who were expected to have extensive knowledge of plants used for food and medicine were selected with the help of the corresponding village committees. Six focus group discussions were conducted in the Daur Autonomous Township, during which healers, ethnic medicine gatherers, and local village committees helped facilitate an understanding of the current situation regarding MEPs among the Daur. This information included the plant resource, daily food consumption, disease treated, ethnic culture, and other relevant information.

### Data analysis

#### Expression correlation network

Topological relationships among MEPs, their health benefits and therapeutic uses, and attending disease classifications were analysed using the Cytoscape 3.9.1 software (https://cytoscape.org/) to obtain key factors and visualise the network [25]. Then, using the plug-in ‘cytoNCA’, we analysed the hub targets, which had a high betweenness centrality value, and chose the top five targets for each MEP, health benefits, therapeutic uses, and body system classification as hub nodes.

#### Informant consensus factor

The informant consensus factor (ICF) represents the general use of plants; it is calculated using the following formula:

$${\text{ICF}} = \, \left( {{\text{N}}_{{{\text{ur}}}} - \, N_{t} } \right) \, / \, \left( {{\text{N}}_{{{\text{ur}}}} - \, 1} \right),$$

where N_ur_ refers to the number of use reports for a particular disease category and N_t_ refers to the number of taxa used for a particular use category by all informants. Low ICF values (close to 0) are obtained if medicinal resources are chosen randomly or if there is no exchange of information about their use among informants. High ICF values (close to 1.0) are obtained when the informants indicate well-defined selection criteria for the species regarding a specific illness category or if the informants are in full agreement regarding the use of a certain species for a specific use [26]. Higher ICF values indicate a stronger agreement among information providers as to which medicinal plants are used in traditional therapies for a particular health issue.

#### Use value (UV) for individual species

The UV—a quantitative measure of the relative importance [27–29]—was calculated for all species in the study area using the following formula:

$${\text{UV }} = \, \sum {\text{U}}_{{\text{i}}} /{\text{N}},$$

where $\sum {\text{U}}_{{\text{i}}}$ is the total number of use reports cited by each informant for a given MEP and N represents the total number of informants. Low UVs (close to 0) indicated few reports related to use, and higher UVs indicated a higher number of reports of MEP use, thus implying species importance.

#### System dynamics (SD) model

Based on the influence of sustainable resources, culture, and socioeconomic factors, the feedback and interactions between subsystems and variables in the MEP development status and the future development trend were analysed to design an SD model. Using the VENSIM software as a modelling tool, a qualitative SD simulation was performed to model the feedback relationships of effect variables to supplement qualitative information gathered from experts and the Daur [30]. The model process relied on systems thinking, that is, the ability to gain understanding by engaging in processes based on mental models [31, 32].

## Results

### Sociodemographic characteristics of the study participants

The MEP use surveys consisted of semi-structured interviews with a total of 122 people in six focus group discussions. The study informants were divided into groups according to age as follows: < 30, 31–40, 41–50, 51–60, and > 60 years old. Most informants were in the 51–60 (51.6%), 41–50 (20.5%), and 31–40 (14.8%) age groups, a distribution that was representative of the main users of ethnic medicine and knowledge inheritors. Few individuals in the youngest (< 30 years old; 5.7%) and oldest (> 60 years old; 7.4%) age categories were interviewed (Table S2).

### Plant biodiversity in the Daur minority area

The field survey collected data in the Ewenki Autonomous Banner, the Arun Banner, Zhalantun City, and the Molidawar Daur Autonomous Banner for several years. A total of 501 species of plants, which belonged to 87 families and 284 genera, were thoroughly studied and recorded. Asteraceae was the dominant plant family used (75 species), followed by Rosaceae (37 species), Ranunculaceae and Leguminosae (32 species each), and Lamiaceae (30 species).

### Diversity of MEPs used by the Daur

In total, 52 species of commonly used MEPs from 29 families and 47 genera were identified using the UV (Table 1). Among them, Rosaceae was the dominant plant family used (seven species), followed by Asteraceae (six species), Amaryllidaceae (four species), Ranunculaceae and Lamiaceae (three species each), and Betulaceae, Cucurbitaceae, Apiaceae, Urticaceae, and Poaceae (two species each). The remaining 19 families (Salicaceae, Cannabaceae, Polygonaceae, Amaranthaceae, Grossulariaceae, Euphorbiaceae, Araliaceae, Ericaceae, Solanaceae, Plantaginaceae, Viburnaceae, Liliaceae, Asphodelaceae, Typhaceae, Fabaceae, Zingiberaceae, Brassicaceae, Rhamnaceae, and Phrymaceae) were represented by one species each. Herbs (39 species, 75%) were the commonly used MEP type, followed by trees (seven species, 13.5%) and shrubs (six species, 11.5%). In the past, most MEPs were collected from the natural environment, but now, with the increasing use of herbal medicines for different purposes, the extent of cultivation has increased, and therefore, approximately 42.3% of these MEPs can be obtained through cultivation.

**Table 1:** List of frequently used MEPs by the Daur in Inner Mongolia, China

Scientific name	Local name	Family	Parts used	Health benefits and therapeutic uses	Body system	Taxonomic	Route, dose and usage	Resource	UV	Voucher Specimens
*Populus tremula* L	Aolihlurudan	Salicaceae	Bark	Dental disease	A	Tree	Or: The water boiled in the bark is used to gargle	Wi	0.1	150724LY0032
*Betula pendula* subsp. *mandshurica* (Regel) Ashburner & McAll	Gaalbaan	Betulaceae	Bark, tree sap	Bacillary dysentery, diarrhoea, gastric ulcer, trauma	A,B,E	Tree	Or: The burnt ashes of the bark are used for brewing. It is roughly 5 g each time, three times a day Ex: Dip a piece of paper in the tar produced by the burning of the bark or part of the branch, and wrap it to the affected area afterwards	Wi	1.07	150724LY0689
*Corylus heterophylla* Fisch. ex Trautv	Xuxgeer	Betulaceae	Seed	Diarrhoea, loss of appetite, cough	A	Tree	Or: Take a decoction of 30-60 g of seeds. Or it can be used in a powdered form	Wi or Cu	0.77	150783LY0367
*Cannabis sativa* L	Har danga daayaan	Cannabaceae	Seed	Smallpox	B	Herb	Ex: A mixture of the same number of seeds and the leaves of *Raphanus sativus* is smashed, and the mucus formed is repeatedly rubbed on the body until the patient sweats	Wi or Cu	0.23	150783LY0331
*Urtica cannabina* L	Malu	Urticaceae	Whole plant	Rheumatoid arthritis, ringworm	C,G	Herb	Ex: Pounded fresh Whole plant and applied to the affected area. Alternatively, the dried whole herb is pounded with sesame oil or boiled in water and washed the affected area	Wi	0.75	150783LY0194
*Urtica dioica* var. *holosericea* Fr	Malus	Urticaceae	Whole plant	Rheumatoid arthritis, ringworm	C,G	Herb	Ex: Mash fresh Whole plant or dry Whole plant with sesame oil and apply to the affected area	Wi	0.75	150721LY0027
*Fagopyrum tataricum* (L.) Gaertn	Haul	Polygonaceae	Seed	Cholera, dyspepsia	A,B	Herb	Or: The seeds are cooked with milk	Wi or Cu	0.99	150783LY0244
*Chenopodium album* L	Kerel	Amaranthaceae	Whole plant	Diarrhoea, dysentery	A,B	Herb	Or: Take a decoction of 15 g of Whole plant	Wi	0.31	150783LY0320
*Actaea dahurica* (Turcz. ex Fisch. & C.A.Mey.) Franch	Zaokeri	Ranunculaceae	Rhizome	Mumps	B	Herb	Ex: Dried rhizomes boiled in water washed the affected area. Or mashed freshly rhizomes to wrap	Wi	1	150783LY0280
*Aconitum kusnezoffii* Rchb	Bonga	Ranunculaceae	Root	Rheumatism	C	Herb	Or: Use 3–6 g of root in decoction or in pills and powder (if used in soups, prepare 1–2 h in advance to minimise toxicity) Ex: Apply wine or vinegar infused with roots to the affected area	Wi	0.73	150722LY0150
*Pulsatilla chinensis* (Bunge) Regel	Qurquu korsuu ilgaa	Ranunculaceae	Root, flower	Rheumatism	C	Herb	Or: Take a decoction of 30-60 g of roots Ex: Pounded fresh flowers wrapped to joints.Or apply wine infused with dried or fresh flowers to the affected area	Wi	0.89	150724LY0298
*Brassica juncea* (L.) Czern	Harik nugua	Brassicaceae	Seed	Arthralgia	F	Herb	Ex: Mix fresh seed powder with egg white and apply to affected area	Cu	0.15	150721LY0117
*Ribes* *diacantha* Pall	Eulunku	Grossulariaceae	Fruit	Cold	D	Shrub	Or: Take a decoction of 6-9 g of fruits	Wi	0.25	150722LY0195
*Crataegus pinnatifida* Bunge	Humpur	Rosaceae	Fruit	Dyspepsia	A	Tree	Or: A small amount of the fruit is taken after a meal	Cu	1	150783LY0089
*Dasiphora* *fruticosa* (L.) Rydb	Altan uralagiin	Rosaceae	Leaf	Digestion	A	Shrub	Or: Take a decoction of 6 g of leave	Wi	0.21	150783LY0229
*Malus baccata* (L.) Borkh	Huliir	Rosaceae	Fruit	Tracheitis	D	Tree	Or: Eat the fruits in winter	Wi	0.3	150722LY0006
*Potentilla flagellaris* D.F.K.Schltdl	Zheltu taolyin tangai	Rosaceae	Whole plant	Gaseous abdominal distention, urethritis	A,J	Herb	Or: Take a decoction of Whole plant	Wi	0.15	150721LY0088
*Prunus padus* L	Moli	Rosaceae	Leaf, fruit	tender leaf: parasites; fruit: enteritis, diarrhoea	A,B	Tree	Or: Fresh leaves or fruit to eat	Wi	0.54	150783LY0368
*Rosa davurica* Pall	Jaam	Rosaceae	Root nodule	Rheumatoid arthritis	C	Shrub	Or/ Ex: The root nodules are boiled into water for drinking and washing the affected area	Wi	0.74	150724LY0166
*Sorbaria sorbifolia* (L.) A.Braun	Subudlig qiqig	Rosaceae	Branch	Costal chondritis, trauma	E,F	Shrub	Ex: The branches are boiled into water for drinking and washing the affected area	Wi	0.92	150721LY0148
*Astragalus membranaceus var. mongholicus* (Bunge) P.K.Hsiao	Mongol huangqii	Fabaceae	Root	Gout	H	Herb	Or: The roots are cooked with barley and nuts	Wi or Cu	0.42	150724LY0071
*Euphorbia fischeriana* Steud	Unie mek	Euphorbiaceae	Root	Tuberculosis, tuberculosis of bones, several kinds of scabies	B,G	Herb	Or: Decoct 100 g of jujubes mixed with 50 g of dried roots, 2 pieces each time, eaten twice a day	Wi	0.38	150722LY0020
*Ziziphus jujuba* Mill	Sor	Rhamnaceae	Fruit	Hepatocirrhosis	A	Tree	Or: The fruits are steamed with *Perilla frutescens* seeds and sugar and eaten	Cu	0.52	150783LY0169
*Cucurbita pepo* L	Weegee tabsag tosbei	Cucurbitaceae	Seed	Ascariasis, disease due to nematode	B	Herb	Or: Eat about 15 g of raw seeds	Cu	0.67	150721LY0147
*Cucumis sativus* L	Mood kenk	Cucurbitaceae	Seed	Fracture	E	Herb	Or: Eat about 15 g of raw seeds	Cu	0.39	150722LY0294
*Panax ginseng* C. A. Mey	Orwoidaa	Araliaceae	Root, rhizome	All kinds of disease	-	Herb	-	Wi or Cu	0.61	150783LY0248
*Cicuta virosa* L	Morii angool	Apiaceae	Root	Venomous snakes or insects bite, extravasation of blood, osteomyelitis, gout, rheumatism	C,E,H	Herb	Ex: Decocted or ground rhizome wrapped to the affected area, mixed with egg white or Cicuta virosa to use together	Wi	0.04	150722LY0247
*Saposhnikovia divaricata* (Turcz. ex Ledeb.) Schischk	Daor kaxienku	Apiaceae	Root, seed	Root: rubella itching, wind-cold headache. Seed: urethritis, hernia	G,H,J	Herb	Or: Take a decoction of 6 g of roots. Or you can boil the seeds directly or with brown sugar as a tea	Wi or Cu	1	150721LY0158
*Rhododendron dauricum* L	Nandig yilega	Ericaceae	Flower, fruit	Tracheitis, pneumonia, irregular menstruation, amenorrhe, uterine bleeding, rheumatism, trauma	C,D,E,H,I	Shrub	Or/ Ex: Fresh flowers 5 g are taken for decoction or extracting in wine. Fruits 2–3 g ground into a powder	Wi	0.72	150722LY0005
*Leonurus japonicus* Houtt	Gentgelj mowoi eus	Lamiaceae	Whole plant	Irregular menstruation, abdominal pain with blood stasis	I	Herb	Or: Boil the flowering whole herb to make a paste. The paste is then taken with water	Wi or Cu	0.78	150722LY0217
*Perilla frutescens* (L.) Britton	Bai	Lamiaceae	Seed oil, seed, leaf	Abnormal sputum, cough, constipation, hepatitis	A,D	Herb	Or: The leaves can be taken after boiling or frying. The seeds can be mashed and taken. Seed oil is applied to the surface of the skin	Wi or Cu	0.93	150783LY0145
*Scutellaria baicalensis* Georgi	Qiee ilgaa	Lamiaceae	Root	Abdominal pain, diarrhoea, dysentery, cough	A,B,D	Herb	Or: The roots are steeped in hot water and consumed as a tea	Wi or Cu	0.89	150721LY0159
*Nicotiana tabacum* L	Danga	Solanaceae	Leaf	Carbuncle, furuncle, dermatitis, eczema, trauma	B,E,G	Herb	Ex: Take a decoction of 6-9 g of leave. Or pounded fresh leave wrapped to the affected area	Cu	0.61	150722LY0287
*Phryma nana* Koidz	Hadal baagii eus	Phrymaceae	Whole plant	lumbago and leg pain	E	Herb	Ex: The Whole plants are boiled with *Saposhnikovia divaricata* root and *Artemisia argyi* leaves, then mixed with vinegar and fumigated with hot air	Wi	0.18	150783LY0358
*Plantago asiatica* L	Morii toroo	Plantaginaceae	Seed	Lumbago and leg pain	F	Herb	Or: Fry the seeds and take 3-9 g of them	Wi or Cu	0.68	150721LY0054
*Sambucus williamsii* Hance	Gaawui mood	Viburnaceae	Branch	Trauma, costal chondritis	E,F	Shrub	Or/ Ex: Fresh branches are boiled and taken internally or scrubbed	Wi	0.27	150783LY0039
*Achillea alpina* L	Tagyin tulegeq ebus	Asteraceae	Whole plant	Appendicitis	A	Herb	Or: Take a decoction of 15 g of Whole plants	Wi	0.56	150724LY0724
*Artemisia argyi* H.Lév. & Vaniot	Suagi	Asteraceae	Whole plant	Urticaria, rheumatism	C,G	Herb	Ex: The whole herb is boiled in water and smoked to wash the affected area or taken orally	Wi	1	150722LY0219
*Artemisia integrifolia* L	Kumbil	Asteraceae	Whole plant	Dysentery, diarrhoea, laryngeal carcinoma	A,B,K	Herb	Or: Take a decoction of Whole plant with beans	Wi	1	150783LY0293
*Filifolium sibiricum* (L.) Kitam	Xrihegelig xierilej	Asteraceae	Whole plant	Pneumonia	D	Herb	Or: A decoction of 3 g of the whole herb and 30 g of the leaves of Rhododendron dauricum are boiled together and taken twice a day	Wi	0.27	150721LY0048
*Jacobaea* *cannabifolia* (Less.) E. Wiebe	Alagalige geiqigna	Asteraceae	Whole plant	Asthma, cough	D	Herb	Or: Take a decoction of 15 g of Whole plants	Wi or Cu	1	150721LY0071
*Xanthium strumarium* L	Jangoo	Asteraceae	Fruit	Rheumatism	C	Herb	Or: Take a decoction of fruits	Wi	0.52	150721LY0164
*Lilium pensylvanicum* Ker Gawl	Jaowees	Liliaceae	Bulb	Cough	D	Herb	Or: The bulbs are steamed, dried and ground into a powder. Patients take 10-15 g with honey water when needed	Wi	0.41	150721LY0116
*Hemerocallis minor* Mill	Gilooq	Asphodelaceae	Root	Suppurative mastitis	I	Herb	Ex: Pounded fresh roots wrapped to the affected area	Wi	0.36	150783LY0074
*Allium fistulosum* L	El	Amaryllidaceae	Leaf sheath	Acute gastroenteritis,stomach flu	A	Herb	Ex: Apply the film from the leave sheath wrap to the anus	Cu	0.64	150722LY0063
*Allium ramosum* L	Guagus	Amaryllidaceae	Leaf	Pediatric nosebleed	D	Herb	Ex: Mash the fresh leaves and drop the resulting juice inside your nose	Wi	0.26	150783LY0044
*Allium sativu*m L	Suaandaa	Amaryllidaceae	Bulb	Chronic nephritis, glomerulonephritis, blood in urine	J	Herb	Or: Cook the pig stomachs and bulbs with bulbs together to serve	Cu	0.37	150722LY0208
*Allium senescens* L	Mangie	Amaryllidaceae	Leaf	Anorexia, weakness and lethargy	A	Herb	Or: Fresh or dried leaves are taken internally in a soup with crucian carp	Wi	0.45	150783LY0254
*Avena sativa* L	Heerkualimp	Poaceae	Seed	Loss of appetite, constipation	A	Herb	Or: The seeds are decocted with water. Or boil the seeds with oats, rice bran and add barley sugar to the rice soup	Cu	0.99	150722LY0029
*Setaria italica* (L.) P.Beauv	Narim	Poaceae	Seed oil	Several kinds of ringworm and scabies	B	Herb	Ex: Apply seed oil to the injured area 2–3 times a day	Cu	0.36	150783LY0192
*Typha orientalis* C.Presl	Xuedaga	Typhaceae	Whole plant	Rheumatoid arthritis, rheumatism	C	Herb	Ex: Heat fresh Whole plant and fumigate the patient for about 1 h	Wi	0.06	150721LY0136
*Zingiber officinale* Roscoe	Ganjiang	Zingiberaceae	Root	Gout	H	Herb	Or: Boil the root with salt and baking soda and soak the feet	Cu	0.25	150722LY0212

The 10 kinds of body systems that may be targeted by MEPs are classified as follows: (A) digestive system, (B) Infectious or parasitic, (C) Rheumatic immunity system, (D) Respiratory system, (E) Trauma, (F) Musculoskeletal system or connective tissue, (G) Skin, (H) Endocrine system, (I) Gynaecological disease, (J) Genitourinary system and (K) Tumour. Or: Oral, Ex: External. Wi: wild, Cu: cultivated

**Fig. 3:**
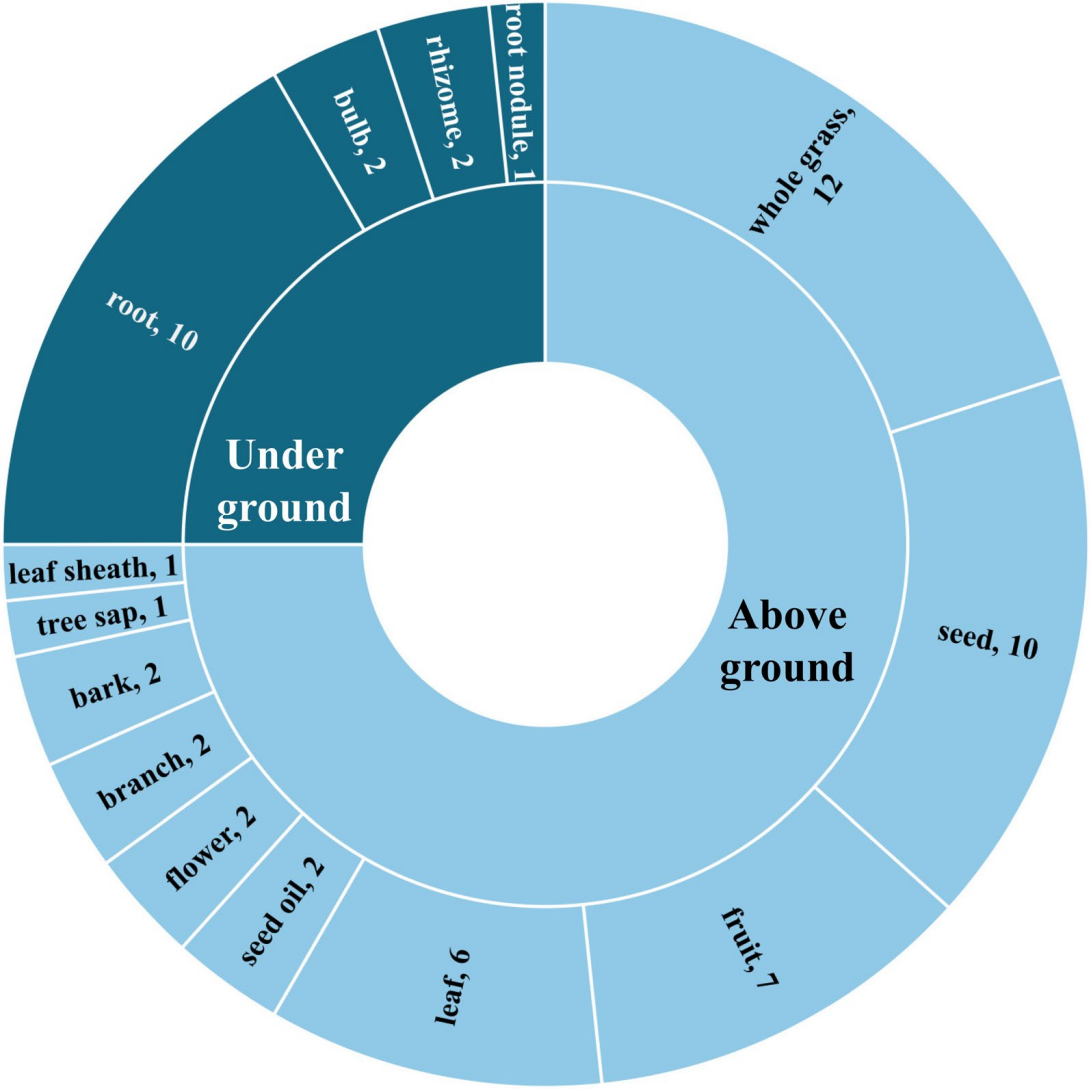
The statistics of the used parts for MEPs of Daur ethnic group

Depending on the plant, various parts may be used in the treatment of diseases. According to the informants, the most commonly used parts are as follows: all parts of the plant (12 species), followed by the root and seed (10 species each); fruit (7 species); leaf (6 species); bulb, rhizome, bark, branch, flower, and seed oil (2 species each); and root nodules, leaf sheath, and tree sap (1 species each) (Fig. 3). Notably, the Daur people predominantly used easily accessible and regenerating aboveground parts of MEPs, which differs from the approach used in traditional Chinese medicine. This largely balances the use of MEPs with the conservation of natural resources.

We also collected information on the administration route, preparation, dosage, and usage of MEPs by the Daur people. According to the present study, MEPs were prepared via decocting, smashing, boiling, extracting, and burning. They were most often ingested as pills or powder or cooked with ingredients such as milk, honey, wine, vinegar, oats, sesame oil, egg white, rice bran, beans, barley, nuts, the crucian carp, or a pig stomach. Some MEPs were used externally in the form of a wash, fumigate, or wrap. Dosages were estimated for most MEPs and were dependent on the age of the patient, severity of the illness, diagnosis, and experience of the healer.

### Use value

The Daur group with ethnobotanical knowledge on their application considered MEPs to be primarily suitable for single therapeutic uses or health benefits (36%), followed by two aspects (34%) and three or more aspects (30%). The quantitative UV indices revealed that the MEPs most used as medicines are *B. pendula* subsp. *mandshurica* (with a UV of 1.07), *Artemisia integrifolia*, *Actaea dahurica*, *Crataegus pinnatifida*, *Saposhnikovia divaricata*, *Artemisia argyi*, and *Jacobaea cannabifolia* (all with UVs of 1.00) and *Fagopyrum esculentum*, *Avena sativa*, and *Perilla frutescens* (with a UV of 0.99, 0.99, and 0.93, respectively). Many parts of these plant species, which are readily available or are typical species in the area, are used as a health food and a medicine.

### Informant consensus factor

The ICF was used to identify plants of particular intercultural relevance and evaluate how homogenous the obtained information was. Overall, 11 disease categories were identified. The ICF was calculated for each disease category, and it ranged from 0.93 to 1.00. The highest ICF (1.00) was obtained for tumour, which involved one species and one use report. However, the high ICF value for this classification may be related to the low number of cases. The classification with the second highest ICF was for rheumatic immunity system problem (0.99) with 5 species and 534 use reports. The high values achieved in the study probably indicate a high degree of consensus among the informants. The classification with the third highest ICF was for skin problem (0.98) with 6 species and 305 use reports, where the use of medicinal species was random, and no consensus was reported among the informants (Table S4).

### Health benefits and therapeutic uses of MEPs by the Daur

**Fig. 4:**
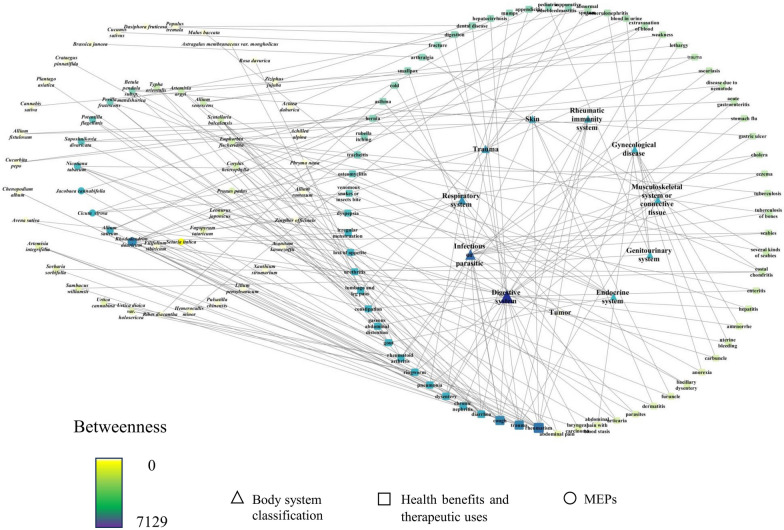
Network visualisation of the relationships between health benefits, therapeutic uses, body system classification and MEPs

Table 1 presents MEPs utilised by the Daur for 85 therapeutic uses; these were classified into 11 groups based on the intended body system or targeted health category. The availability of the network model structure may improve our understanding of the relationships between MEPs, health benefits, therapeutic uses, and medical classification. According to the network statistics, the number of nodes was 129, the number of edges was 194, and the average node betweenness was 416 (Fig. 4). Statistically, the ‘Digestive system’ category was most frequently detected and included 22 health benefits and therapeutic uses, including those for dental disease, diarrhoea, gastric ulcer, loss of appetite, cough, dyspepsia, gaseous abdominal distention, enteritis, hepatitis, abdominal pain, dysentery, laryngeal carcinoma, rheumatism, weakness, lethargy, acute gastroenteritis, and stomach flu. Other categories included ‘Infectious or parasitic’, ‘Rheumatic immunity system’, ‘Respiratory system’, and ‘Trauma’. Treatment for rheumatism, trauma, cough, diarrhoea, and chronic nephritis were among more important health benefits and therapeutic uses. The health benefits and therapeutic use classification indicated a link between traditional MEP knowledge and modern medicine; however, the MEP knowledge of the Daur ethnic group retained its inherent characteristics. Among the uses, various common ailments (cough, constipation, diarrhoea, and trauma) and endemic diseases (rheumatism and rheumatoid arthritis) were treated with numerous therapies as part of the basic health service provision (Table 2).

**Table 2:** Top 5 nodes of MEPs, health benefits, therapeutic uses, and body system classification in the network ranked by the betweenness centrality value in “cytoNCA”

Rank	MEPs (Betweenness Centrality)	Health benefits and therapeutic uses (Betweenness Centrality)	Body system classification (Betweenness Centrality)
1	*Allium sativum* (791)	rheumatism (3774)	Digestive system (7129)
2	*Cicuta virosa* (509)	trauma (2811)	Infectious or parasitic (4278)
3	*Jacobaea cannabifolia* (504)	cough (2808)	Rhododendron dauricum (2737)
4	*Nicotiana tabacum* (425)	diarrhoea (1122)	Respiratory system (2265)
5	*Saposhnikovia divaricate* (396)	chronic nephritis (1013)	Trauma (1793)

### Relationship among cultural, socio-economic, and resource factors with MEPs

The SD model method considers the dynamic interactions among multiple factors and thus meets the requirements of simulation systems and can more clearly represent changing relationships. We present a model with four closely related subsystems: resources, culture, socioeconomic factors, and MEP use. Flowcharts demonstrating the relationships among and between the variables of each subsystem are shown in Fig. 6. The resources’ subsystem includes resource protection, exploitation, and overexploitation. This subsystem describes the interrelationship among various elements of sustainable resource use. The culture subsystem includes education quality. Higher education systems often negatively affect the spread of ethnic languages and religious beliefs, but promote further communication of the scientific literature. The socioeconomic subsystem includes urbanisation, large-scale farming, economic growth, and the population. This subsystem mainly describes how changes in societal norms and economic development affect the major production modes and lifestyle traits of individuals within the population. The negative ( +) and positive (-) markers associated with each arrow indicate the direction of influence of each factor on the others. The series of positive and negative causal relationships among the three subsystems and the use of MEPs form the causal feedback loops of this study.

## Discussion

### Regional variations in MEP knowledge in inner Mongolia and Xinjiang

**Fig. 5:**
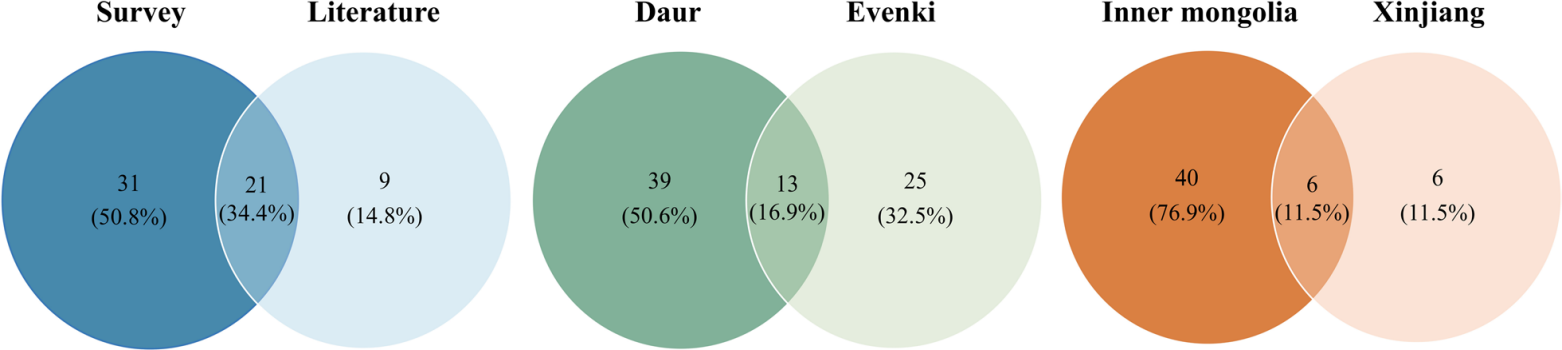
Comparison of MEP species between regions and ethnic groups

There were regional differences in the MEPs used by the Daur ethnic group. Reporters from Inner Mongolia have cited a total of 52 plant species, and those from the Xinjiang region have cited 12 species (Fig. 5). Six shared species between the regions (*S. divaricate*, *P. frutescens*, *Allium sativum*, and *A. argyi*) and native (*Urtica cannabina* and *Phryma nana*) plants were cultivated. The species overlap between the Xinjiang and Inner Mongolia regions was minimal in the MEP use survey, which may be related to differences in the living environments and lack of inheritance of knowledge. In our investigation in Tacheng, we found just two elderly people who carried out traditional Daur medical practices, and they mainly used interventional therapies, such as treating concussion by tapping a rope tied to the patient's head. We gathered information on national costumes, festivals, and diets from Daur museums, but literature on the use of MEPs was scarce. The MEP use among the Daur ethnic groups in the Xinjiang region was mostly dependent on cultivated species that were readily available, including *Ziziphus jujuba* and *Zingiber officinale* used as food seasonings. An important reason for this is also the serious loss of ethnobotanical knowledge within the Daur ethnic group in Xinjiang, which greatly reduced the information collected during the investigation process.

### Differences in knowledge of MEPs between the Daur and Ewenki ethnic groups

In previous studies, our team collected information on Ewenki medicine in the same region of Inner Mongolia [22, 33]. Medicinal information was collected on 38 species of MEPs, among which 13 species were common and their usage was duplicated between the ethnic groups, with similar methods and therapeutic applications. The Ewenki ethnic group utilised 25 kinds of unique medicinal plants. The Daur and Ewenki people share areas of distribution in Inner Mongolia. In addition, the two ethnic groups have historically been in close contact [34], which has led to similarities in the plant resources used and living environments between these ethnic minorities. Comparison of the investigated MEP information in relation to the Daur and Ewenki ethnic groups (Fig. 5) showed that most species were selected from local common wild or planted species. The 13 species used by both ethnic groups included *U. cannabina*, *S. divaricata*, *Plantago asiatica*, *A. argyi*, *A. integrifolia*, *Allium fistulosum*, *A. sativum*, *Rosa davurica*,* Malus baccata*, *Z. jujuba*, *Setaria italica*, *B. pendula* subsp. *mandshurica*, and *Prunus padus.* Among them, *A. integrifolia* was popular among the people from both minority groups and was frequently used as a medicine, in the daily diet, and during festive celebrations. However, owing to the differences in ethnic heritage, there were some significant differences in the use of MEPs between the two ethnic groups.

The data collected in this survey of MEP resources differed significantly from previous records of medicinal use in the Daur. This difference is mainly reflected in the fact that this survey is about the application of MEPs in the current living environment, rather than simply recording past use reports. In this process, it was tried to understand the changes in the application of MEPs under the influence of different ethnic groups and different regions. Thus, we observed that the medical practices of the Daur ethnic minority are related to access to resources; the species currently used are relatively easy to obtain from wild or cultivated resources, and the increased diversity of plant types can be attributed to the exchange of knowledge with different ethnic groups.

### MEP resources and ecological sustainability

Our investigation in Inner Mongolia revealed that the MEPs used by the Daur communities accounted for ~ 10% of all plant resources in the minority nationality area. Owing to their distribution and habitat status, herbs are more frequently used as medicines and food than woody plants. In addition, the Daur communities have shown a preference for perennial wild plants and cultivated species over annuals for medicinal and edible use. Aerial parts, seeds, and fruits are easier to obtain than underground parts and are more palatable for both medicinal and edible use in daily life. It was evident that plant selection depended on ecological factors, empirical factual knowledge, and living habits, in addition to MEP use characteristics gained from experience.

**Fig. 6:**
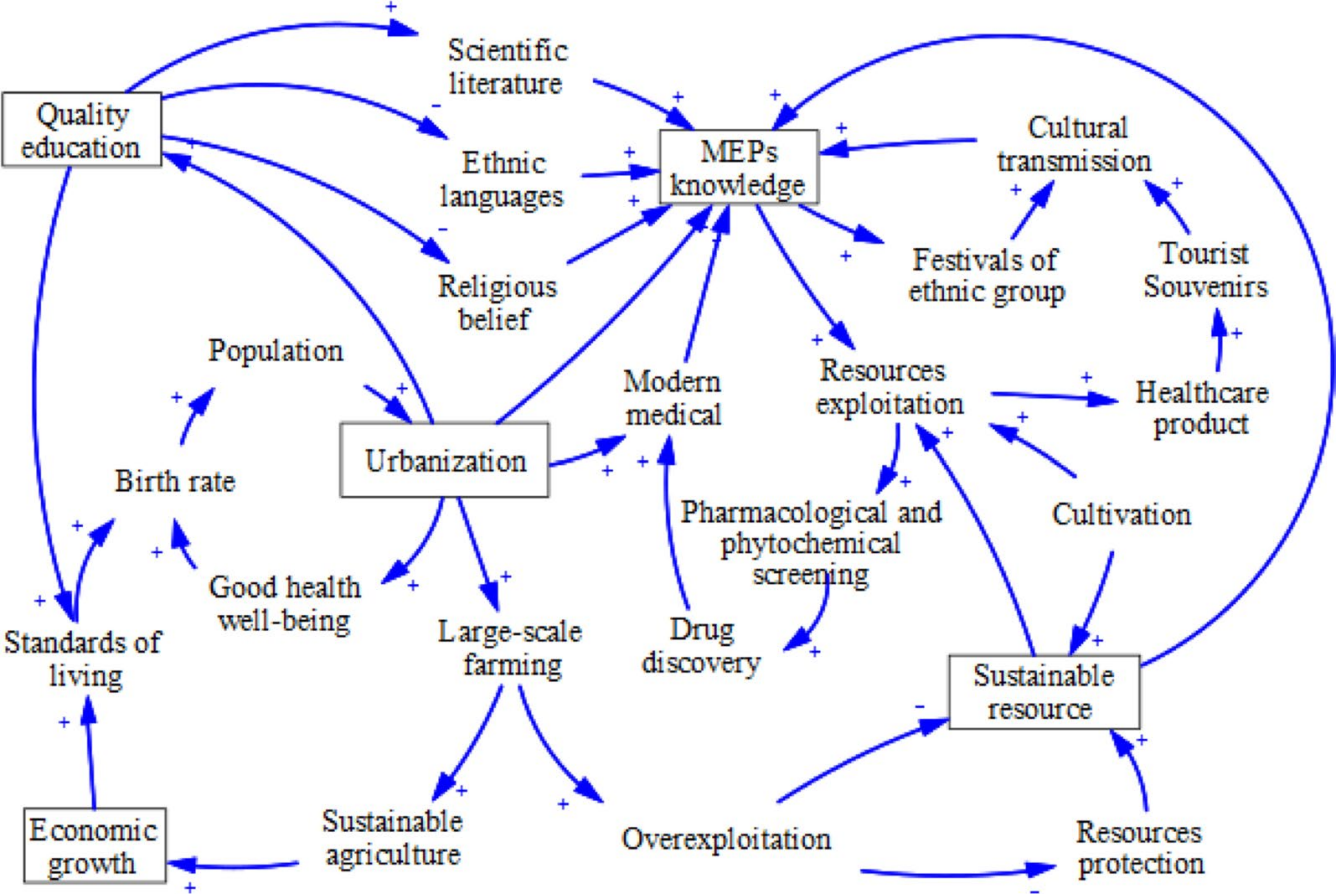
System dynamics flowchart of MEPs exploitation

The major regions inhabited by the Daur ethnic group are rich in natural resources. These insights into the distribution of MEPs will aid in the conservation and management of local ecosystems, biodiversity, and endangered species habitats. Currently, some studies have suggested that the climate change will affect biodiversity in certain regions at a faster rate than other factors in the coming decades [35, 36]. The ongoing climate change increases the probability of local conditions becoming warmer and wetter, consequently making the environment more hospitable to native species and suitable for cultivation of other species. Although the climate change has played a role in improving resource abundance, we identified potential negative effects of overcollection, overgrazing, and deforestation on MEP sustainability. Strengthening forest management and conservation has protected the environment in many cases, whereas access to wild MEP resources has become more limited. Connecting MEP use and resource conservation with a local policy, cultural diversity, and biodiversity is critically important for sustaining the socioecological resilience and traditional medicinal systems of indigenous people (Fig. 6). If information on MEP use can infiltrate local ethnobotanical knowledge through text and media communications, MEPs will likely be better developed and utilised.

### Cultural protection of Daur MEPs

The use of traditional Daur MEPs holds great cultural value, representing traditional knowledge and the utilisation of nature in Daur society. The Daur ethnic group in Inner Mongolia lives in the Greater Khingan Mountains region, where there are extensive birch forests. In historical hunting and gathering society, the Daur people used the bark of the *B. pendula* subsp. *mandshurica* to make a variety of utensils and furniture, which we now only see in museums and souvenir shops [37]. However, the formation of the *B. pendula* subsp. *mandshurica* culture had an invisible impact on people who used it, and it has a high use value. The traditional knowledge of the indigenous Daur people on the medicinal usage of plants, emphasising the deep connection between humans and nature. The Daur people regard *A. integrifolia* as a national cultural symbol. The Chinese name for *A. integrifolia* is ‘Kumule’. The ‘Kumule Festival’ was historically established to honour the significance of *A. integrifolia* in the survival of the Daur. In the festival, groups of Daur individuals pick willow sprouts to make traditional foods, and gather together. *A. integrifolia* has gradually developed into an important cultural symbol and has been recorded in many characters, promoting the spread of national culture [38]. But plants that combine cultural, medicinal, and edible attributes are still in the minority, so most people tend to be more limited in their knowledge of medicinal and edible plants.

Interviews and meetings with Daur communities revealed that Daur traditional MEP knowledge was mainly limited to herbalists and older people, with less knowledge held by younger generations. Traditional MEP knowledge is often transmitted through oral traditions and practical experience, and a reduction in carriers of this ethnobotanical knowledge could result in a diminished understanding and effective utilisation of natural resources. According to historical records, the Daur people have had a high level of education and became urbanised early [39]. As a result, their close relationships with the traditional natural environment through production modes and lifestyles, such as fishing, hunting, and animal husbandry, as well as dietary and medical aspects, have diminished or weakened. Actively recording knowledge on the traditions and uses of MEPs and properly preserving this information will be conducive to knowledge protection and inheritance. In addition, supporting the research, production, and promotion of ethnic minority medicine through government subsidies and project funding will increase the dissemination of Daur ethnic knowledge in local social applications.

Geographically related groups share MEP resources and knowledge, but the uniqueness of each group's traditions within their specific environmental and cultural contexts is preserved. When comparing regional and ethnic differences, we found that changes in any one of these elements could have imposed an unknown, unquantifiable influence on MEP use. Deeper insights into the characteristics and values of different MEPs will provide broader perspectives and support for their preservation and inheritance.

### MEP resource development

The Daur ethnic group has centuries of successive experience in acquiring MEP knowledge and in MEP use. Most MEPs used by the Daur people are readily available in the local environment, in addition to many cultivated herbals that constitute a considerable part of the medicinal flora. Specifically, *A. integrifolia* is a well-known local wild plant species used in medicine to treat various diseases, including hypertension, hyperlipidaemia, diabetes, and hepatitis [21]. The Daur have used this species as an important part of their food system. Previously, *A. integrifolia* was used to improve predominantly meat-based diets to reduce the risk of bowel cancer. The species is now considered a healthy diet staple and is used in soups and other dishes as part of daily meals [38]. *P. frutescens* is another well-known species among the Daur; the leaves and seed oil are included in Daur diets, in addition to the use of fruits, seeds, and seed oil in health care. One important medicinal property of *P. frutescens* is that its seed oil is rich in alpha-linolenic acid, which has many benefits for human health. The species has been cultivated over an area > 2000 hm^2^ within the Oroqen Autonomous Banner. In-depth knowledge of the traditional uses of medicinal plants can provide clues for modern drug discovery and development. Many modern drugs have origins traced back to plants used in traditional medicine, so preserving and studying these traditional uses can contribute to discovering new therapeutic approaches.

The present study revealed that future changes in traditional culture and socioeconomic environments would likely result in the loss of traditional medicine preferences, as this valuable natural asset is restricted to indigenous cultures. Globalisation and cultural exchanges can lead to conflicts between different cultures, impacting the transmission of traditional medicine. In addition, some cultures may be perceived as more modern or advanced, leading to disdain or neglect of traditional medicinal practices. Therefore, we should fully document the valuable MEP knowledge of the Daur ethnic group. An additional solution is to increase MEP use in daily edible consumption. In China, this phenomenon of decreased use of traditional medicines is gradually becoming more common among the small number of ethnic minorities who retain a large body of MEP-related knowledge but lack dispersed spoken and written languages. Advances in modern medicine have led to the widespread use of pharmaceutical therapies, which are often more accessible, standardised, and, in some cases, more effective. People are increasingly inclined to choose modern medicine over traditional practices, reducing the demand for traditional plant-based remedies. MEPs with significant health benefits and therapeutic uses should be explored in future studies, with a particular focus on their conservation, pharmacological mechanisms, and phytochemical properties. Additionally, products such as perilla seed oil, honey, and tea have been developed for nutrition and health care and are often sold as tourist souvenirs.

To address these challenges, comprehensive measures are necessary, including the promotion of cultural preservation, support for the transmission of ethnobotanical knowledge, encouragement of sustainable resource utilisation, and the establishment of an integrated healthcare system that incorporates both modern and traditional medicine. This can be achieved through policy-making, community engagement, and education.

## Conclusion

With the rise of modern medicine and rapid socioeconomic development, the ethnic cultural heritage and traditional ethnic medicines, particularly discovery the use of MEPs, are gradually lost. However, if traditionally used MEPs are increasingly incorporated into modern lifestyles for daily health maintenance, this may improve dietary species diversity. To broaden the role of MEPs in daily lives of global populations, we conducted research on the MEPs used by the Daur communities of Inner Mongolia. Through interviews, focus groups, and fieldwork, we collected physical specimens and knowledge related to 52 species of MEPs that are characteristically used to remedy regional diseases and for daily health care. Identify the important species as highlighted by the ICF and UVs. We designed an SD model to show the relationships between sustainable resources, culture, and socioeconomic factors. Ethnic medicinal research is an ongoing process, during which more potent MEPs and a rich phytochemical diversity can be gradually discovered and applied in multiple settings and situations. As we increase our utilisation of wild resources to improve dietary diversity, nutrition, and health care, we must simultaneously assess and manage these resources to ensure their sustainability in accordance with the Convention on Biological Diversity and local protection strategies.

### Supplementary Information

Supplementary file1

## Data Availability

The datasets used and analysed during the current study are available from the corresponding author on reasonable request.

## Abbreviations

MEP: Medicinal and edible plant
UV: Use value
ICF: Informant consensus factor
SD: System dynamics
