# Supplementary material for: An ethnobotanical survey on the medicinal and edible plants used by the Daur people in China
Source: J Ethnobiol Ethnomed. 2024 May 24;20:55. doi: 10.1186/s13002-024-00695-8 (PMC11127305; doi:10.1186/s13002-024-00695-8)
Supplement: Supplementary file 1 — Supplementary file1 [file 13002_2024_695_MOESM1_ESM.docx]

**SUPPLEMENT**

**Table 1** Visited locations

|  | Ewenki Autonomous Banner | Arun Banner | Zhalantun City | Molidawar Daur Autonomous Banner | Tacheng City |
| --- | --- | --- | --- | --- | --- |
| GPS | W: 47°32'50″-49°15′37″  E: 118°48'02"-121°09'25" | W: 47°56'54"-49°19'35"  E: 122°02'30"-124°05'40" | W: 47°5′40″-48°36′34″  E: 120°28′51″-123°17′30″ | W: 48°05′10″-49°50′50″  E: 123°32′55″-125°16′14″ | W: 43°25′-47°15′  E: 82°16′-87°21′ |
| Altitude | 580-1690 m | 160-1160 m | 140-1670 m | 160-950 m | 1100-5000 m |
| Area | 19111 km2 | 13641 km2 | 16785 km2 | 10356 km2 | 105000 km2 |
| Number of inhabitants | 134790 | 316772 | 396980 | 308202 | 145000 |
| Ethnicity | 23 ethnic groups, including Han, Mongolian, Daur and so on | 24 ethnic groups, including Han, Manchu, Daur and so on | 28 ethnic groups, including Han, Mongolian, Daur and so on | 27 ethnic groups, including Han, Mongolian, Daur and so on | 29 ethnic groups, including Han, Hui, Daur and so on |
| Language | Chinese-based | Chinese-based | Chinese-based | Chinese-based | Chinese-based |

**Table 2** Socio-demographic characteristics of the informants

| Basic information | Number of informants | Percentage (%) |
| --- | --- | --- |
| Sex |  |  |
| Male | 68 | 55.7 |
| Female | 54 | 44.3 |
| Age |  |  |
| ≤30 | 7 | 5.7 |
| 31-40 | 18 | 14.8 |
| 41-50 | 25 | 20.5 |
| 51-60 | 63 | 51.6 |
| ＞60 | 9 | 7.4 |

**Table 3** Questionnaire

| No. | Question |
| --- | --- |
| 1 | Name of the participant. |
| 2 | Participant’s age and gender. |
| 3 | Address of the participant. |
| 4 | Interview date. |
| 5 | How long do you live in the given area? |
| 6 | Local name of the used plant. |
| 7 | Which diseases are treated by the plant? |
| 8 | Which part is used? |
| 9 | What is the method of remedy preparation? |
| 10 | What is the approximate dose? |
| 11 | What are the problems affecting the development of Daur medicine？ |

**Table 4** Informant consensus factor for ailment categories

| System categories  (group of illness) | Number of use reports (Nur) | % of use reports | Number of taxa (Nt) | % of taxa | Informant consensus  factor (ICF) |
| --- | --- | --- | --- | --- | --- |
| Digestive system | 957 | 27.20 | 22 | 25.58 | 0.97 |
| Infectious or parasitic | 553 | 15.71 | 16 | 18.60 | 0.97 |
| Rheumatic immunity system | 534 | 15.17 | 5 | 5.81 | 0.99 |
| Respiratory system | 411 | 11.68 | 9 | 10.47 | 0.98 |
| Trauma | 274 | 7.79 | 8 | 9.30 | 0.97 |
| Musculoskeletal system or connective tissue | 134 | 3.81 | 4 | 4.65 | 0.97 |
| Skin | 305 | 8.67 | 6 | 6.98 | 0.98 |
| Endocrine system | 107 | 3.04 | 5 | 5.81 | 0.96 |
| Gynecological disease | 162 | 4.60 | 6 | 6.98 | 0.96 |
| Genitourinary system | 50 | 1.42 | 4 | 4.65 | 0.93 |
| Tumour | 32 | 0.91 | 1 | 1.16 | 1 |
| Total | 3519 |  | 86 |  |  |
